# Review of Guideline Recommendations for Optimal Anti-VEGF Therapy in Age-Related Macular Degeneration

**DOI:** 10.3390/life14101220

**Published:** 2024-09-24

**Authors:** Andreea Dana Moraru, Ciprian Danielescu, Raluca Eugenia Iorga, Radu Lucian Moraru, Mihail Zemba, Daniel Constantin Branisteanu

**Affiliations:** 1 Department of Ophthalmology, ‘Grigore T. Popa’ University of Medicine and Pharmacy, 700115 Iași, Romania; oftalmoconsultiasi@yahoo.ro (A.D.M.); daniel.branisteanu@umfiasi.ro (D.C.B.); 2 Department of Ophthalmology, ‘N. Oblu’ Clinical Hospital, 700309 Iași, Romania; 3 ‘Transmed Expert’ Medical Center, 700011 Iași, Romania; 4 Department of Ophthalmology, ‘Dr. Carol Davila’ Central Military Emergency University Hospital, 010825 Bucharest, Romania; 5 Department of Ophthalmology, Railway Clinical Hospital, 700506 Iași, Romania

**Keywords:** age-related macular degeneration, anti-VEGF therapy, treat and extend, intraretinal fluid, subretinal fluid

## Abstract

Neovascular age-related macular degeneration is a progressive, blinding macular disease that has become a burden both in healthcare systems and the global economy. The vascular endothelial growth factor (VEGF) is the main agent involved in the pathogenic process of the disease. The main goal of the age-related macular degeneration treatment is to maintain and improve visual acuity by injecting intravitreal anti-VEGF agents in either a reactive or proactive manner. Subretinal and intraretinal fluids are the main biomarkers that should be considered when managing the frequency of the therapy. This review discusses both functional and morphological treatment criteria according to current recommendations as opposed to real-life situations encountered during day-to-day clinical practice and highlights situations in which the benefits of continuing therapy are arguable in terms of improving patients’ quality of life. Optimizing the treatment regimen represents an important aim of current clinical ophthalmological practice, as age-related macular degeneration patients usually have a long follow-up period.

## 1. Introduction

Neovascular age-related macular degeneration (nAMD), or macular neovascularization as it is currently known (MNV) is a progressive, chronic, blinding retinal disease affecting 8.7% of the world population [1]. One meta-analysis taking into account the European population reported a prevalence of early or late AMD of 0.08% in the group of 50- to 55- year-old participants, which increased with age to a percent of 20.1% in the group of participants over 90 years old [2]. It is estimated that 10% of patients diagnosed with the non-exudative form of age-related macular degeneration eventually develop the exudative form [3]. Visual impairment caused by MNV has become a burden to both the global economy and the overly crowded healthcare system.

The main pathological event in MNV is the formation of a choroidal neovascular membrane (CNV) that penetrates the Bruch membrane, leading to exudation in the subretinal space, retinal edema, hemorrhage, pigment epithelial detachment, and subretinal fibrous scars. Clinical and experimental data have demonstrated that vascular endothelial growth factor (VEGF) is the main agent involved in inflammatory and angiogenetic process [4,5].

VEGF is a neurovascular trophic factor important for the function of both endothelial cells and neurons. VEGF activity is important for sustaining nervous tissue, the cardiovascular system, and the renal system. The eye also intervenes in the development and normal function of the retinal vasculature [6]. VEGF plays an important role in the expression of nitric oxide by endothelial cells; thus, inhibition of VEGF causes dysfunction of the vascular endothelium and may lead to thromboembolic events, systemic hypertension, and renal impairment [7].

Understanding of the physiopathological mechanism has facilitated the emergence of new therapeutic methods. Thus, over the last two decades, several anti-VEGF agents have been studied and approved as first-line treatments for nAMD. Current therapeutic protocols are under continuous evaluation in order to improve the results obtained in real--life treatment of patients with nAMD.

The current review aims to bring into discussion the therapeutic protocols of the available anti-VEGF agents, the main biomarkers useful in guiding the therapy, the risk factors associated with the treatment, and some future directions that may influence clinical practice in the following years.

## 2. Therapeutic Protocols and Available Drugs

There are currently several drugs that can be used to treat neovascular age-related macular degeneration.

Bevacizumab was the first drug used, off-label, in nAMD therapy. It was administered by intravitreal injection and showed major improvements in visual function and retinal morphology of nAMD patients [8,9].

Ranibizumab is a recombinant humanized immunoglobulin (Ig) G monoclonal antibody fragment that binds to VEGF A, thus inhibiting its effects. It is smaller than a full-size antibody and penetrates the retina with ease, but it is cleared from the vitreous quickly, with a half-life of only 9 days [10]. Its efficacy in maintaining vision was demonstrated in two double-blind studies, ANCHOR and MARINA, over the course of two years. The ANCHOR study also demonstrated that ranibizumab is superior to photodynamic therapy for the treatment of classic neovascular membranes [11].

Aflibercept is a widely used VEGF Trap-Eye, which is a chimeric protein containing the second binding domain of the VEGFR-1 receptor and the third domain of the VEGFR-2 receptor. It has high affinity for all isomers of VEGF A, VEGF B, and placental growth factor. The VIEW 1 and VIEW 2 studies demonstrated its non-inferiority for visual function maintenance compared to ranibizumab [12,13].

Two other drugs were recently approved for the treatment of macular neovascularization: Brolucizumab and faricimab. Brolucizumab is a humanized monoclonal single-chain variable fragment; thus, it is the smallest functional unit of an antibody that binds and inhibits VEGF A at a ratio of 2:1 [14]. Two studies, HAWK and HARRIER, demonstrated its non-inferiority to aflibercept in maintaining visual acuity and its superiority in reducing intraretinal and subretinal fluid as compared to the same drug [15].

Faricimab is a bispecific antibody that binds to both VEGF A and Angiopoietin 2. Its efficacy was demonstrated in two comparative studies, TENAYA and LUCERNE, which showed similar visual function gains between the group treated with aflibercept and those receiving faricimab. The results were maintained throughout the second year of treatment, with the possibility of extending the dosing interval for faricimab to 8, 12, or 16 weeks, depending on the functional and anatomic results [16].

The main goal of MNV treatment is to maintain and improve visual acuity by injecting anti-VEGF agents such as aflibercept, brolucizumab, ranibizumab, and bevacizumab in the vitreous cavity. The treatment can be administered in either a reactive or proactive manner but should be individualized in order to obtain optimal results.

The reactive approach, or pro re nata (PRN), means that injections are administered only when the disease shows signs of activity, such as intraretinal or subretinal fluid. This type of approach reduces the number of injections to which the patient has to be submitted but, requires very rigorous monitoring in order to capture the moment in which a dormant CNV resumes activity. Although patients do not follow a monthly injection regimen, they need to follow a monthly examination schedule, which enables the ophthalmologist to determine if retreatment criteria are met based on clinical, OCT (optical coherence tomography), and angiographic assessment. Recent literature data has shown that PRN usually leads to suboptimal results due to delayed or low-frequency dosing [17,18].

Treat-and-extend (T and E) is a proactive type of therapeutic management for MNV. In T and E, the patient has to follow a regular schedule of injections, starting with an implementation phase, in which the injections are administered monthly, and continuing with a maintenance phase, when the interval between injections is lengthened gradually until the morpho-functional aspect of the disease becomes stable. The advantage of this type of therapeutic approach is that it minimizes the risk of undertreatment or overtreatment of delayed administration and reduces the number of hospital visits compared with a monthly regimen of injections, which does not consider whether the disease shows signs of activity.

**Figure 1:**
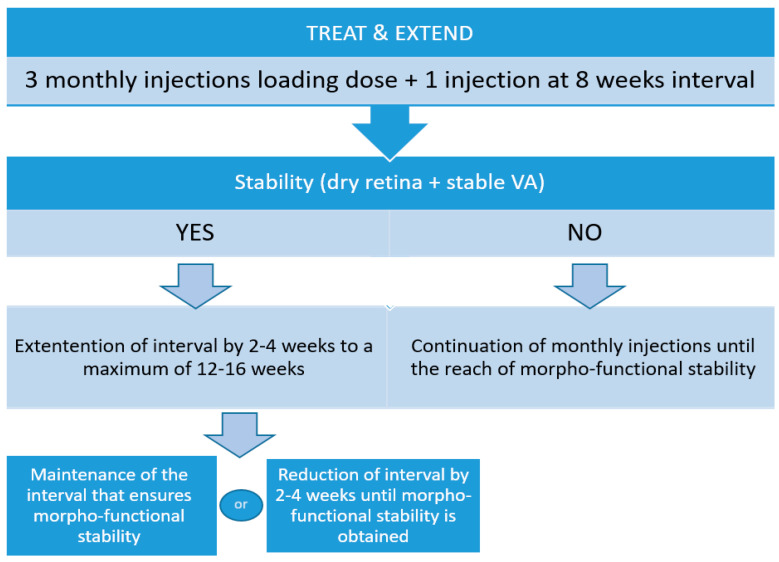
Treat-and-extend algorithm for intravitreal injections in neovascular AMD. Morpho-functional stability is defined as the loss of no more than five ETDRS letters in visual acuity and no fluid or no new fluid on OCT.

Currently, the treatment recommendations for intravitreal anti-VEGF treatment are to perform a loading dose, consisting of three-monthly doses, followed by another injection at eight weeks intervals [17]. If, at this point, the disease becomes stable based on morpho-functional criteria (visual acuity and OCT aspect), the period of time to the 5th injection can be extended by 2 to 4 weeks, to a maximum of 12 weeks. Similarly, the interval to the next injection can be extended to a maximum of 16 weeks if the disease is stable and there are no signs of disease progression. The interval of time should be maintained if changes in visual acuity and OCT morphology occur when a longer period of time between injections is being implemented. However, if visual acuity worsens due to loss of more than 5 ETDRS letters and OCT shows signs of disease activity consisting of subretinal and intraretinal fluid and macular hemorrhages, then the interval between injections should be reduced by 2 to 4 weeks until disease inactivity is achieved (Figure 1).

Newer studies have shown that an increase in the administered anti-VEGF dosage could lead to relaxation of the therapeutic protocol, with the possibility that after the loading phase, the interval between two intravitreal injections might be lengthened to 8 to 12 weeks. Over the same period, high-dose aflibercept produced better anatomical results, with a greater number of eyes free of macular fluid and no compromises regarding the safety of administration, as compared to standard-dose aflibercept. Despite the better morphological results, the difference between the final visual acuity was not statistically significant between the groups treated with aflibercept 2 mg and 8 mg [19,20].

## 3. Biomarkers of Treatment Response

Subretinal and intraretinal fluids are the main biomarkers that should be taken into account when managing the T and E regimen. (Table 1) Visual acuity should also be considered in the analysis, but it is not a specific biomarker for disease activity like the morphological changes seen on OCT, as it can be influenced by several other factors, such as cataract progression.

**Subretinal fluid (SRF)** should be treated until the disease becomes stable and the subretinal space is dry. Sometimes, despite correct and frequent treatment, a small quantity of SRF persists. Recent studies have shown that a small and stable quantity of SRF can be safely tolerated by patients without the need to increase the number of injections. The exact volume of SRF considered tolerable should be determined by the supervising physician. The stability of SRF levels and absence of new morphological changes are key factors in the management of these cases. Both the CATT and FLUID studies showed no significant difference in visual acuity at 1 year follow-up between patients with and without a small amount of SRF. The FLUID study demonstrated that visual acuity at 1 year was similar in those who followed a T and E dosing protocol aimed at eradicating all SRF and those who received fewer injections and tolerated a fluid volume of less than 200 µm [21,22].

A retrospective analysis after 1 year of treatment with aflibercept showed that the gain in visual acuity was similar in patients with inactive and active disease, defined by the presence of SRF, intraretinal cysts, and intraretinal fluid and that after the loading dose, the macular status could predict the results obtained at 1 year interval [23].

The occurrence of pigment epithelial detachment, hemorrhage, and modifications of the choroidal neovascular membrane, together with the persistence or addition of new SRF, are signs of retreatment necessity.

Currently, there is no consensus regarding the extent to which residual SRF can be considered tolerable. Recently, an artificial intelligence-assisted analysis of patients enrolled in the FLUID study stated that an SRF-tolerant treat-and-extend regimen was associated with worse functional prognosis. Extended intervals between injections, despite the presence of residual SRF, were associated with higher volumes of SRF in the central 6 mm area of the macula [24].

**Intraretinal fluid (IRF)** represents a sign of disease activity, and it is associated with a lower visual acuity than SRF, both at baseline and after treatment. CATT study showed that at both 1 and 5 years of treatment, the gain in visual acuity was reduced in patients with IRF compared with those without IRF [10]. IRF present at baseline correlates with disease activity and is a sign of poor prognosis even in treatment-naïve patients [25].

IRF can cause morphological alterations in the retina due to the loss of photoreceptors. The presence of IRF for a long time may determine cystic degeneration of the retina over areas of atrophy and fibrosis [26]. This is why many eyes do not improve significantly in visual acuity despite following a strict protocol of treatment.

Two recent studies used artificial intelligence technology to analyze OCT data and correlate the quantity and disposition of the SRF and IRF with the functional benefit obtained after anti-VEGF therapy [27,28].

Schmidt-Erfurth et al. demonstrated in these studies that the presence of IRF is the most important biomarker for predicting visual acuity regression, while the influence of SRF is much weaker. The presence of SRF in the juxtafoveal area also depends on volume, but not in the central foveal region, which is associated with a decrease in visual acuity. The IRF has a reverse association, as its presence in the central fovea and, to a lesser extent, in the juxtafoveal area is associated with poorer visual function [27].

IRF should not be confused with outer retinal tubulation. **Outer retinal tubulation** is a retinal feature associated with degeneration, in which the photoreceptors are rearranged in a circular pattern associated with degenerative diseases of the retinal tissue, including age-related macular degeneration, dystrophies, etc. [29].

This lesion is non-specific and needs to be differentiated from an IRF for proper management. On optical coherence tomography (OCT) images, outer retinal tubulation can be distinguished from an intraretinal cyst by its hyperreflective borders surrounding a hyporeflective space located in the outer nuclear layer [30].

This aspect is due to the loss of the adhesion of the photoreceptors, which fold inward with the outer limiting membrane. The tubular structure protects the photoreceptors from injuries as they are located in the lumen. The structure is located at the border between an atrophic lesion and normal retinal tissue, where the retinal pigment epithelium is absent or altered [31].

The outer retinal tubulation is non-responsive and does not require anti-VEGF treatment, which is a factor of reserved visual prognosis [32].

**Sub–retinal pigmentary epithelium (sub-RPE) fluid,** usually described on OCT as pigmentary epithelium detachment (PED), seems to have a less important impact on the visual function prognosis. In the VIEW and HARBOR studies, the patients who were diagnosed with PED had a higher visual acuity at baseline than those without, but the association was weaker during the follow-up period. At 2 years follow-up, the eyes with better visual acuity at baseline maintained superior visual function compared to the rest [33]. The presence of foveal PED was associated with better visual acuity at 5 years in the CATT study, as sub-RPE fluid may have a trophic effect on the retina [34].

However, eyes that presented with complete resolution of PED at 24 months had a higher risk of developing macular atrophy [35].

The integrity of the **foveal receptor layer** is another biomarker with important implications for visual prognosis. The disruption of the ellipsoid zone and the external limiting membrane band negatively affects the outcome of anti-VEGF therapy [36,37,38].

However, the correlation between the integrity of these layers and the visual acuity appears to be stronger at baseline assessment than after VEGF therapy [39].

**Hyperreflective dots** are retinal lesions scattered throughout all layers, but with a preferred disposition around the cystoid spaces, with a higher reflectivity than the RPE. They are thought to be formed by lipid-filled microglia and migrated RPE cells [40].

The presence of hyperreflective dots has negative prognostic value. These lesions are associated with lower visual acuity at baseline and their persistence inside the retinal layers is correlated with worse visual function after treatment [41].

**Subretinal hyperreflective material**, located between the RPE and the neurosensory retina, is thought to consist of fibrin, blood, fluid, scar, or macular neovascularization and is associated with a lower visual acuity, regardless of its disposition and with less improvement in visual function after treatment [42]. Its composition can change over time. If the subretinal hypereflective material has a neovascular component, which can be identified by OCT angiography, then the response to anti-VEGF therapy is usually weak [43]. One study suggested that if reflectivity decreases after treatment, the visual prognosis could be better [44].

Recently, a higher incidence of **vitreomacular interface alterations**, in particular, vitreomacular adhesions and vitreomacular traction, has been described [45]. This correlation seems to be caused by the development of the neovascular membrane with its exudative and fibrotic phases, making the adhesions between the posterior hyaloid and the retina stronger [46]. These eyes tend to respond poorly to anti-VEGF therapy and require a greater number of intravitreal injections [47].

Vitreomacular traction may cause alteration of the inner and outer retinal layers and lead to chronic inflammation, which may accelerate the progression of MNV [48]. Eyes with vitreomacular traction might benefit from surgical removal of traction by reducing the number of intravitreal injections needed to stabilize the neovascular membrane [49].

Some **alterations in choroidal morphology** may also be biomarkers that influence the outcomes of intravitreal anti-VEGF therapy. These include sub-RPE hyperreflective columns, prechoroidal clefts, choroidal caverns, subfoveal choroidal thickness, and the choroidal vascular index [50].

Sub-RPE hyperreflective columns are a sign of broken RPE, which may open the path for blood, fluid, and neovessels to enter the subretinal space [51].

Prechoroidal clefts appear in eyes with chronic fibrovascular and multilayered PED as hyporeflective cavities between the choroid and the fibrous component of the PED. They usually have a spindle shape and are surrounded by hyperreflective lamella with contractile properties. The exudation and contraction of the sub-RPE neovascular membrane cause delamination of the RPE−Bruch’s membrane complex and of the choroid. These eyes maintain good visual acuity because the lesion is located under the RPE and responds to anti-VEGF therapy [52]. Another explanation for the preservation of relatively good visual function is the possible nutritional effect of the sub-RPE complex on the outer retinal layers, thus protecting the retina from geographic atrophy [53]. Due to its morphology, the prechoroidal cleft is associated with a lower risk of developing RPE tears.

In AMD, the choroidal caverns may be associated with neovascular membranes or geographic atrophy. They appear as hyporeflective sub-RPE structures visible on en-face and cress-sectional OCT. They are thought to be either non-perfused ghost vessels at the site of a previous neovascular membrane or lipid-rich Friedman globules, and do not require treatment [54,55].

The influence of subfoveal choroidal thickness and choroidal volume on visual prognosis and treatment outcomes is still under debate. Choroidal thickness can be assessed by swept-source OCT, and it is influenced by circadian rhythm, injected anti-VEGF drug volume, and systemic pressure [56].

However, choroidal hypoperfusion is considered a risk factor for the development of AMD. Several studies have reported a reduction in the choroidal vascular index in relation to the progression of AMD lesions toward neovascular membranes [57].

The choroidal vascular index is defined as the area of the choroidal vascular lumen within the area of the choroidal stroma. A reduction in this index correlates with choroidal hypoxia [58].

## 4. Signs of Choroidal Neovascular Membrane Reactivation

Despite a good response to therapy and achievement of disease stability, a significant number of cases reactivate over a variable period of time. After following the T and E protocol, the risk of reactivation was higher when the interval between injections was longer. One-fifth of the patients that were treated at 16 weeks intervals suffered from recurrence of symptoms [59].

New signs of CNV activity include recurrence or increased amount of SRF and/or IRF, subretinal hemorrhage, new CNV, or pigmentary epithelium detachment that was not present in previous examinations. These cases need to be treated immediately either by reducing the interval between injections by 2–4 weeks or by beginning a new loading phase with 3 monthly injections, depending on the gravity of lesions observed on OCT. Switching between anti-VEGF agents may also be an option [17].

## 5. Non-Responsive Cases

Non-responsiveness to treatment is usually associated with subretinal fibrosis present in the final stage of the disease or the development of atrophic changes in the retinal pigmentary epithelium (RPE) and the photoreceptor layer.

The reduced therapeutic effect from the outset may be due to a diagnostic error, most likely due to confusion between CNV and polypoidal choroidal vasculopathy or retinal angiomatous proliferation. The involvement of VEGF in the pathogenesis of polypoidal choroidal vasculopathy is less important than in MNV; thus, the response to anti-VEGF therapy is limited. Encouraging morpho-functional results have been obtained with aflibercept treatment [60].

Retinal angiomatous proliferation is considered a type 3 neovascularization with a suboptimal response to anti-VEGF therapy [61].

The pathogenesis of retinal angiomatous proliferation is both ischemic and inflammatory, with multiple growth factors involved in the development of lesions, accounting for the limited response to intravitreal treatment.

Studies based on genetic testing have concluded that there are some genetic variants of MNV that are less responsive to anti-VEGF therapy and more prone to frequent recurrences [62]. Future treatment guidelines might have to take into account an individualized treatment regimen based on genetic variants.

There is a subgroup of patients who show either minimal or no response to therapy from the outset. Morphological assessment of these eyes shows persistence of intraretinal, subretinal, or sub-RPE fluid and sometimes additional hemorrhages. The unresponsiveness of these eyes is due to chronic alterations in the retinal tissue, RPE, and fibrosis. This may also be due to an exudative syndrome other than MNV.

Chronic central serous chorioretinopathy may present with subretinal and sub-RPE fluid and chronic cystoid degeneration of the retinal tissue that is similar to MNV. Polypoidal choroidal vasculopathy (PCV) is part of the neovascular proliferation spectrum, but it has different characteristics from MNV and is usually less responsive to anti-VEGF injections. This pathological entity is part of the pachychoroid spectrum of diseases, which has been shown to respond poorly to intravitreal therapy [63]. Nevertheless, several studies (EVEREST, LAPTOP, PLANET, EPIC) have shown that both functional and morphological results are better when PCV is treated with intravitreal anti-VEGF therapy, either as monotherapy or in combination with photodynamic therapy [64,65,66,67].

Neovascular membranes that develop as a result of an inflammatory disease tend to be less responsive to anti-VEGF agents because the VEGF pathway is not the only pathway responsible for the pathogenic process. The use of steroids and immunosuppressive agents may be beneficial in these cases [68].

The development of drug tolerance is another cause of poor treatment response. The inflammatory cells that are located in the neovascular membrane respond to anti-VEGF therapy by upregulating the number of VEGF receptors and the production of VEGF. At the same time, the immune system’s response to the drug, and the secretion of neutralizing antibodies, accelerates the clearance of the injected substance from the eye. Both the ANCHOR and MARINA studies described an increase in immunoreactivity to anti-VEGF after the first year of monthly administration of the drug [69,70].

In theory, an increase in the dosage or frequency of administration may overcome poor response to therapy. Several studies, including the HARBOR trial, demonstrated that a higher dose of anti-VEGF does not lead to a better morpho-functional result in all patients [71,72,73].

The HARBOR study compared outcomes following the administration of either a standard 0.5 mg dose of ranibizumab or a 2 mg dose of the same drug. This study found no statistically significant differences between the two groups in terms of anatomical and functional outcomes in treatment-naïve patients. Two other studies, LAST and SAVE, demonstrated a minor improvement in visual acuity and morphological results at 6 months when treatment-resistant patients were switched from a 0.5 mg dose to a 2 mg dose of ranibizumab [74,75].

Increasing the frequency of administration may also improve morpho-functional outcomes. This is due to the higher levels of anti-VEGF achieved by increasing the frequency of administration compared to increasing the dose. The maximum reduction in the neovascular membrane size was achieved 12 to 18 days after treatment [76].

While twice-monthly dosing may improve immediate morphological outcomes, it may also open the door to more aggressive disease regression with the development of more SRF and IRF and decreased patient compliance [77].

Tachyphylaxis to anti-VEGF agents was considered a possible cause of the lack or insufficient response to therapy despite following a strict protocol. Tachyphylaxis is quantified by assessing the improvement in central retinal thickness and was defined in a recent study as no reduction or worsening of this parameter after at least two monthly injections [78].

Hara et al. observed that tachyphylaxis to anti-VEGF agents was more likely to occur in eyes with neovascular lesions under the RPE than in eyes with occult CNV and polypoidal choroidal vasculopathy but not in eyes with intraretinal edema [78].

One possible approach to tachyphylaxis is to switch anti-VEGF agents to take advantage of the superior binding affinity or pharmacokinetics of another drug [79,80,81].

Since the introduction of aflibercept, many studies have analyzed outcomes after replacing ranibizumab or bevacizumab with aflibercept [82,83].

Most of these studies reported an improvement in macular morphology but less benefit in visual acuity [84,85].

In some cases, anti-VEGF treatment not only does not bring the expected morpho-functional improvement, but is also accompanied by worsening visual acuity and a higher quantity of subretinal fluid. This is due to the development of an RPE tear, most likely a result of CNV membrane contraction after anti-VEGF therapy. The incidence of RPE tears is approximately one-fifth of cases treated with anti-VEGF agents, which is higher than the number of tears associated with the natural evolution of a CNV membrane [86]. Several risk factors for the development of an RPE tear have been identified, such as a large pigmentary epithelium detachment, both in height and basal diameter, recent, multilobular or fibrovascular pigmentary epithelium detachment, and smaller size of the CNV membrane by comparison to the detachment size [87]. The imminent development of an RPE tear may be indicated by the appearance of wrinkling of the RPE surface visible on OCT examination or radial lines visible on the infrared image. The presence of these signs may be an indication of treatment postponement. After tear development, fluid leakage may be greater due to the absence of the RPE, but the CNV progresses toward fibrosis and, thus, reduction of exudation. Stopping treatment does not appear to offer any benefit, especially if the disease is showing signs of activity. Patients with small tears may experience a small increase in visual acuity if treatment is continued, and patients with large tears may benefit from continued treatment by achieving stabilization and preventing further visual loss [88]. Therapy should be individualized by careful monitoring of these cases. The visual prognosis for these eyes is usually poor, especially if the RPE tear involves the fovea or is associated with subretinal scars or hemorrhage [89].

Another possibility for disease evolution is the sudden onset of massive hemorrhaging under the retina or the RPE. This is most often associated with the rupture of the fragile neo-vessels. Some risk factors include anticoagulant therapy, blood dyscrasias, trauma, Valsalva maneuvers, and more than three months interval between anti-VEGF agent administration [90].

Small hemorrhages may benefit from more frequent anti-VEGF therapy. However, large hemorrhages can be treated by surgical removal of blood but have poor functional prognosis.

The lack of response to anti-VEGF treatment, consisting of persistent exudation after 6 months of monthly treatment, is considered by one expert group to be refractory or treatment-resistant MNV [91]. The same study group classified cases that presented with either recurrence of SRF, IRF, or a new subretinal hemorrhage, despite an initial favorable response to treatment, such as recurrent MNV. Seventy% of patients fall into this category, as they present with a recurrence of symptoms between the first and second years of therapy [92]. Many cases with recurrence of disease tend to have a good response to retreatment, while others develop refractory MNV.

## 6. Anti-VEGF Therapy Ocular Side Effects

The main event associated with long-term anti-VEGF therapy is geographic atrophy. A five-year follow-up of more than 1000 MNV patients treated with intravitreal injections reported an incidence of 12–17% at one and two years and almost 40% after five years of therapy in eyes that were not previously diagnosed with geographic atrophy [93].

The same conclusion is supported by the HARBOR, SEVEN-UP, and IVAN studies [94,95,96].

The follow-up period for the SEVEN-UP study was eight years of ranibizumab treatment and reported a 98% incidence of geographic atrophy detected by fundus auto-fluorescence.

Some experimental studies conducted on rat and primate eyes have shown that intravitreal anti-VEGF treatment generates geographic atrophy as a result of the reduced fenestration of the choriocapillaris during treatment [97].

A few risk factors associated with the development of macular atrophy have been identified: lower baseline visual acuity, reticular pseudodrusen, depigmentation, a lower number of anti-VEGF injections, and retinal angiomatous proliferation [98].

In contrast, the presence of SRF alone, without IRF, seems to be a protective factor against the development of macular atrophy [99,100].

A common event associated with intravitreal anti-VEGF therapy is ocular hypertension. In most cases, the increase in ocular pressure is transient and is related to the volume of drug injected. Approximately 10% of patients who follow intravitreal anti-VEGF treatment for a long period develop chronic ocular hypertension. The risk factors include pre-diagnosed glaucoma, a large number of injections, and a high frequency of intravitreal administration [101].

The pathogenic mechanism is reported to be a decrease in the aqueous humor outflow facility due to the inflammatory effects of anti-VEGF agents on the trabecular meshwork and its direct obstruction by drug particles [102].

The less common adverse effects of intravitreal anti-VEGF therapy include exacerbation of macular ischemia, retinal vascular occlusions, anterior optic neuropathy, and tractional retinal detachment [103].

The OCTOPUS and SWIFT studies analyzed the incidence of ocular inflammatory events associated with brolucizumab treatment in naive patients and in patients previously treated with ranibizumab. Brolucizumab therapy was associated with a higher risk of ocular inflammation than other agents, estimated at 10.5% of the treated patients, of which 3.4% presented with either retinal vasculitis or vascular occlusion, besides vitritis [104]. A few other studies have examined the relationship between brolucizumab therapy and ocular inflammation, suggesting that, in some patients, the anti-VEGF agent triggers an autoimmune response. Although the inflammatory response is mild in most eyes, some have experienced significant vision loss [105,106,107,108].

## 7. When to Stop Treatment

Disease stability is the main criterion for stopping treatment. Recent studies have concluded that it is safe to discontinue the T and E protocol when morpho-functional stability is achieved after two or three intravitreal injections administered at 12-to-16-week intervals [109,110].

The disease should then be monitored three to four times per year, and both visual function and macular morphology should be assessed using OCT, angiography, or OCT angiography. Recurrence should be treated with a monthly regimen followed by an extension of the interval of administration. The success rate of anti-VEGF therapy is greatly influenced by the prevention of recurrences, and such a proactive approach to treatment allows better control of the disease, reduces the likelihood of treatment delays, and helps anticipate and plan the next steps in treatment [111].

## 8. Future Directions in Neovascular AMD

Several studies have used artificial intelligence (AI) to analyze the morphological features of neovascular AMD. Thus, it is possible to predict the evolutionary trend of the disease, risk of exudation, need for therapy, number of intravitreal injections required to achieve stability of the neovascular membrane, visual outcomes obtained with therapy, better choice of treatment, and risk of conversion to geographic atrophy [112,113,114,115,116,117].

Thus, new biomarkers are likely to emerge, and the future of intravitreal therapy is likely to be greatly aided by AI, which can only be seen as an improvement in case management and reduction in the socioeconomic burden of this disease.

## 9. Conclusions

As the number of patients affected by neovascular age-related macular degeneration continues to increase, so does the demand for intravitreal anti-VEGF therapy, which, if properly administered, can put significant strain on the ophthalmic healthcare system. A large number of patients benefit from this treatment, which improves the stability of visual acuity. Strict adherence to the chosen protocol of administration ensures a high success rate in most cases, as long as the selection of cases is based on a thorough follow-up of the main biomarkers that guide therapy. Discriminating cases that benefit from therapy from those that should discontinue it could make a great difference in the quality of medical care offered to patients.

## Figures and Tables

**Table 1:** Correlation between OCT biomarkers and their influence on visual prognosis.

Biomarkers	OCT Aspect	Influence on Visual Prognosis
**Intraretinal fluid (IFR)**	Increased retinal thickness, Fluid cysts above the outer plexiform layer	Signs of disease activity Negative prognostic on VA May determine cystic degeneration of the retina
**Subretinal fluid (SRF)**	Fluid between the RPE and the neurosensory retina	Indicates the need to continue therapy A small and stable quantity may be tolerated without increasing but also without reducing the number of intravitreal injections
**Sub- retinal pigment epithelium (RPE) fluid**	Pigmentary epithelium detachment (PED)	Less impact on visual acuity
**Foveal receptor integrity**	Disruption of the ellipsoid zone and the external limiting membrane band	Negative impact on visual acuity (especially on baseline VA)
**Hyperreflective dots**	Small retinal conglomerates with a reflectivity higher than the RPE, located in all the layers, mostly around the cystoid spaces	Negative prognostic value Correlation with recurrences
**Subretinal hyperreflective material**	Large retinal conglomerates located between the RPE and the neurosensory retina	Negative impact on visual acuity regardless of its disposition and associated with a lower increase of the visual function after treatment
**Vitreomacular interface alterations**		
Adhesion	Strong adhesions between the posterior hyaloid and the retina due to the development of the neovascular membrane	Less responsive to anti-VEGF therapy
Traction	Important traction of the retina exerted by the posterior hyaloid may cause alteration of the inner and outer retinal layers	Less responsive to anti-VEGF therapy Benefit from the surgical removal of the traction
**Alterations in choroidal morphology**		
Sub-RPE hyperreflective columns	Sign of broken RPE Blood, fluid, and neovessels enter the subretinal space	Negative prognostic value
Prechoroidal clefts	Spindle-shaped hyporeflective cavities located between the choroid and the fibrous component of a multilayered PED	Less impact on visual acuity Possible protective effect on the outer retinal layers
Choroidal caverns	Hyporeflective sub-RPE structures	No prognostic value on VA
Subfoveal choroidal thickness	Assessed by SS-OCT High variability	No prognostic value on VA
Choroidal vascular index	The choroidal vascular lumen area reported to the choroidal stromal area	No prognostic value on VA Correlates choroidal hypoxia
**Outer retinal tubulations**	Non-specific lesions in which photoreceptors rearrange in a circular manner Hyperreflective borders surrounding a hyporeflective space located in the outer nuclear layer Located at the border between an atrophic lesion and normal retinal tissue, with the RPE absent or altered	Negative prognostic value

Table 1 summarizes the main biomarkers previously discussed, their morphological aspect on OCT, and the influence each of them has on visual rehabilitation and functional prognosis during therapy. The persistence of intraretinal fluid (IFR), disruption of foveal receptor integrity, presence of hyperreflective dots, subretinal hyperreflective material or sub-RPE hyperreflective columns, and appearance of outer retinal tubulations are highly suggestive of a poor functional prognosis.

## Data Availability

The datasets used and/or analyzed during the current study are available from the corresponding author upon reasonable request.
